# Semantic control across different phenotypes of Alzheimer’s disease: a cognitive study

**DOI:** 10.1002/alz.092396

**Published:** 2025-01-03

**Authors:** Pilar Armas, Sara E Zsadanyi, Alejandra O. Morcillo‐Nieto, Ignacio Illán‐Gala, Sonia Karin Marques‐Kiderle, Francesco Ciongoli, Sara Rubio‐Guerra, Alberto Lleo, Juan Fortea, Stephanie M Grasso, Richard J Binney, Marco Calabria, Javier Hernando, Alexandre Bejanin, Miguel A Santos‐Santos

**Affiliations:** ^1^ Sant Pau Memory Unit, Department of Neurology, Hospital de la Santa Creu i Sant Pau, Biomedical Research Institute Sant Pau ‐ Universitat Autònoma de Barcelona, Barcelona Spain; ^2^ Universitat Politecnica de Catalunya (UPC), Barcelona Spain; ^3^ Sant Pau Memory Unit, Hospital de la Santa Creu i Sant Pau, Biomedical Research Institute Sant Pau, Universitat Autònoma de Barcelona, Barcelona Spain; ^4^ Centre of Biomedical Investigation Network for Neurodegenerative Diseases (CIBERNED), Madrid Spain; ^5^ Faculty of Health Sciences, Universitat Oberta de Catalunya, Barcelona Spain; ^6^ Sant Pau Memory Unit, Department of Neurology, Hospital de la Santa Creu i Sant Pau, Biomedical Research Institute Sant Pau ‐ Universitat Autònoma de Barcelona, Barcelona, Spain Spain; ^7^ Barcelona Down Medical Center, Fundació Catalana Síndrome de Down, Barcelona Spain; ^8^ University of Texas, Austin, TX USA; ^9^ Institute for Cognitive Neuroscience, School of Psychology and Sport Science, Bangor University, Gwynedd United Kingdom; ^10^ Hospital de la Santa Creu i Sant Pau ‐ Biomedical Research Institute Sant Pau ‐ Autonomous University of Barcelona, Barcelona, Catalonia Spain

## Abstract

**Background:**

While the functioning of semantic memory has been extensively documented in Alzheimer disease (AD), little is known about semantic control capacities that monitor and modulate semantic representations. The present study used a task to assess semantic control in typical and atypical AD patients.

**Method:**

11 patients with typical AD (ADtyp), 17 patients with logopenic variant of primary progressive aphasia (lvPPA, i.e. AD language variant), and 16 cognitively unimpaired subjects performed a task (from Corbertt et al., 2011) consisting of selecting the picture of an object that could be used to complete an everyday task (e.g. “kill a fly”). Each task was performed under 4 conditions (4 trials), that manipulated the difficulty of selecting a suitable object. For each trial, the target item was either a canonical object for completing the task (e.g. “flyswat”) or a noncanonical one (e.g. “magazine”); the target picture was presented alongside 5 additional pictures of either unrelated distractors (e.g. “toothbrush”) or distractors semantically related to the target (e.g. “mousetrap”). Semantic control measures were canonicity (associated with abstraction and flexibility capacities) and relatedness (associated with inhibition). Semantic control scores were computed as the difference between canonical and noncanonical conditions (canonicity scores), and unrelated and related conditions (inhibition scores) in accuracy and reaction time (RT); higher scores indicated greater semantic control impairment.

**Result:**

Linear mixed‐model analyses showed that, across all groups, canonicity influenced RT and accuracy, whereas relatedness influenced accuracy only. Interaction analyses showed that the canonicity (but not the relatedness) manipulation resulted in slower RTs (but not lower accuracy) in both patient groups vs controls; lvPPAs unexpectedly showed faster RT in the condition theoretically demanding higher semantic control. Significant differences were found between controls and both patient groups in canonicity scores (both accuracy and RT) but not inhibition scores. No significant differences were observed between the patient groups.

**Conclusion:**

Our results indicate that AD impacts semantic control capacity, with differences for canonicity and inhibition. Canonicity and inhibition semantic control abilities are similarly impaired in lvPPA and ADtyp groups. These results help us to identify different effects of semantic control capacities.

